# The Life-Changing Diagnosis in a Patient With Treatment-Resistant Depression, From Darkness to Light and Healing

**DOI:** 10.1192/bjo.2025.10735

**Published:** 2025-06-20

**Authors:** Saima Jehanzeb, Amna Javaid

**Affiliations:** 1 American Centre for Psychiatry and Neurology, Al Ain Abu Dhabi, UAE; 2 Psychiatry UK Online, UK, United Kingdom

## Abstract

**Aims:** Background: Standard depression treatments often provide partial relief but fail to address the underlying cognitive and motivational deficits associated with undiagnosed comorbid Attention Deficit Hyperactivity Disorder (ADHD), leaving patients feeling stuck in an endless cycle of procrastination, fatigue, and low mood. This case study explores how a missed diagnosis can lead to years of failed depression treatment and how crucial the diagnosis of comorbid conditions in both depression and ADHD is.

**Methods:** Case report.

A 36-year-old health professional presented with chronic insomnia, severe anxiety, low motivation, and persistent depressive symptoms that significantly impacted his professional and academic performance. Despite multiple trials of SSRIs, SNRIs, and antipsychotic augmentation, he continued to struggle with procrastination, difficulty initiating tasks, and performance anxiety, leading to numerous exam failures resulting in low self-esteem, self-doubt, emotional exhaustion, and thoughts of self-harm.

He developed a dependence on clonazepam (4 mg daily) to manage his chronic insomnia, leading to anxiety. This led to cognitive clouding and emotional instability, which exacerbated his struggle with low motivation, attention, and memory difficulties. Insomnia, mind-wandering, forgetfulness, and irritability continued with poor response to medications. An ADHD assessment was conducted using DSM–5 diagnostic criteria, and diagnosis of Adult ADHD Combined Type was confirmed.

The patient was given a trial of Vyvanse (lisdexamfetamine) 30 mg initially and increased to 50 mg once daily, along with venlafaxine 150 mg OD. The introduction of the stimulant brought about a near-immediate improvement in the patient’s condition. He reported increased focus, motivation, and productivity. Procrastination decreased, leading to better academic performance, cognitive clarity, improved sleep, and emotional engagement, resulting in better treatment outcomes. The patient could gradually taper clonazepam and pass his post-graduate clinical exams. The patient responded to a combination of stimulant and antidepressant immediately and described the diagnosis as life-changing. This emphasised further the importance of looking for comorbid conditions while assessing any patient with treatment-resistant depression.

**Results:** Standard antidepressants alone can fail in addressing underlying causes like executive dysfunction and emotional dysregulation associated with undiagnosed Adult ADHD. Robust treatment plans should address both ADHD and any comorbid conditions. Conventional antidepressant treatment proved ineffective until the introduction of Vyvanse and venlafaxine, which highlights the need for ADHD screening in individuals with persistent depression.

**Conclusion:** Comorbid conditions such as depression and anxiety in Adult ADHD can further complicate recognition and proper management, leading to symptom masking and misdiagnosis. The negative impact of undiagnosed and untreated adult ADHD is multifaceted, affecting education, employment, career, and financial stability.

